# How to optimize the benefits of computer assisted sperm analysis in experimental toxicology

**DOI:** 10.1186/1745-6673-8-6

**Published:** 2013-03-11

**Authors:** Carsten Schleh, Anne-Laure Leoni

**Affiliations:** 1 Department of in vivo Pharmacology / Toxicology, BSL BIOSERVICE Scientific Laboratories GmbH, Behringstr. 6 / 8, Planegg/Munich 82152, Germany

**Keywords:** Computer-assisted sperm analysis, CASA, Sperm motility, Reproductive toxicology

## Abstract

Exposure at the working place to various substances, that may affect semen quality is possible and should be investigated in detail. One appropriate method for this is computer-assisted sperm analysis (CASA) which offers multiple benefits in comparison to manual evaluation. However, several pitfalls exist, which make the evaluation of data obtained from CASA difficult to interpret. In the present commentary, we focus on these problems, show some examples, and try to define minimum standards which should be taken into consideration whenever working with computer-assisted sperm analysis.

## Background

Exposure of workers to various substances at the work place may account for observed adverse health effects. One possible effect is an adverse influence on the quality of semen, which has been linked or at least discussed for a number of work place related substances – e.g. nanoparticles, which generally can reach the circulation [1] and even reproductive organs to affect sperms [2], and also metal welding materials [3], or pesticides [4]. Hence, it is of urgent need to investigate the potential adverse effects of substances found in working environments which may affect human semen quality. Although clinical studies deliver the most relevant knowledge, pre-clinical in vivo studies are important to understand the toxicological properties of work place related substances and are often the only possibility to test a substance in complex (in vivo) systems. Several parameters exist to assess male sperm quality and one of the most important is sperm motility [5]. The main methods for assessing sperm motility are manual counting and computer-assisted sperm analysis (CASA). Especially CASA is a powerful tool to investigate sperms and nowadays a lot of scientific laboratories use this technique. Although CASA has several advantages as compared to manual analysis, the complex system is susceptible to false negative or positive results due to inappropriate use. Hence, a thorough validation procedure as well as user training is absolutely necessary in order to profit from these powerful machines. In the following, we discuss the opportunities and pitfalls of CASA to analyze rat sperms in pre-clinical studies, by means of the specific CASA device TOX IVOS Sperm Analyzer from Hamilton Thorne. Furthermore we try to define minimum standards which should be taken into consideration whenever working with computer-assisted sperm analysis in pre-clinical studies.

### Main text

In contrast to manual counting, CASA uses hardware and software to visualize and evaluate consecutive images of viable sperms to obtain precise and valid information on the kinematics of individual sperms. Both methods (manual and CASA) have their benefits and disadvantages (Table 1) but the most promising method is CASA. One disadvantage of manual counting is that an effective use of the hemocytometer is highly dependent on accurate pipetting, dilution, and careful calculation, all of which are common sources of error [6]. Examples for benefits of CASA are fast and detailed objective analysis combined with a high reproducibility, while using identical instrument settings. Various parameters like a smoothed path velocity, track velocity, straight line velocity, amplitude of lateral head displacement, or beat cross frequency can be obtained, and this allows a detailed view into the behavior of individual sperms. Although an enormous amount of data (like the powerful parameter curvilinear velocity) can be generated from even one single measurement, it represents a tremendous opportunity to get a detailed view of the mechanism of adverse effects of a substance, but this amount of information is also a risk because the detailed relevance in prediction the natural conception is at least unclear [7].

**Table 1:** Benefits and disadvantages of manual sperm analysis and computer assisted sperm analysis

**Method**	**Advantages**	**Disadvantages**
Manual analysis	Low acquisition cost	Takes relatively long time for analysis
		Subjective Counting
		Only rough analysis
Computer Assisted Analysis	Fast analysis	High acquisition costs
	Highly reproducible with same settings	Regularly maintenances necessary
	Detailed analysis	Different settings may dramatically change results
	High statistical power due to objective analysis of numerous sperms	

**Figure 1:**
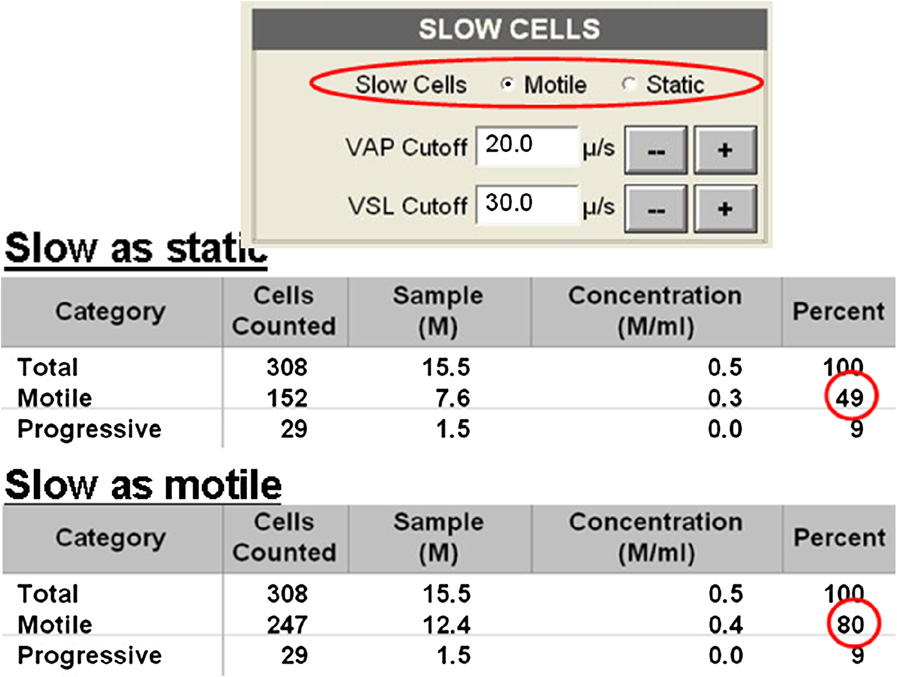
**Analysis of the motility of the same sperm sample with slight changes in instrument settings. **Sperms which were identified as slow sperms may be counted as “motile” or “static”. This slight change by means of a tick in the respective box is able to change the results of the whole study. Remaining sperms were considered as static (data not shown).

Importantly, CASA is sensitive to small changes in the instrument settings which may lead to a profound change of results. For example, a small tick in the box (“Slow sperms are counted as Motile /Static” may lead to completely different results of e.g. an observation of 49% motile cells instead of 80% (Figure 1). Although valid arguments exist for both of possibilities - 1: Slow cells ARE motile; or 2: Slow cells will never reach the oocyte – there is a high risk of misinterpretation of the results for each of the choices; a possibility of an over-interpretation of the respective information due to missing background knowledge. In which groups do the aforementioned slow cells belong?

Another example of an important information is the image acquisition frequency; e.g. 50 Hz and 60 Hz. Different frequencies can have a significant impact on the obtained results [8]. Parameters like this should be considered before buying a specific CASA device and scientific evaluation needs to take these information into consideration.

Only the knowledge of all important details makes it possible for the reader (of a report or publication) to understand scientifically the results of the described study. In this context, each possible individual setting within the software may have its eligibility – especially in the wide field of basic science. It is therefore absolutely necessary to adjust the respective settings in a scientifically valid way, to interpret the results considering the conditions of the individual experiment and hence to assure the maximum scientific output of the study. To ensure this, we suggest that all adjusted settings should be presented in the supplementary information of any future publication. By following this recommendation we can obtain the maximum relevant data for the scientific community and misinterpretations of the results can be avoided.

**Figure 2:**
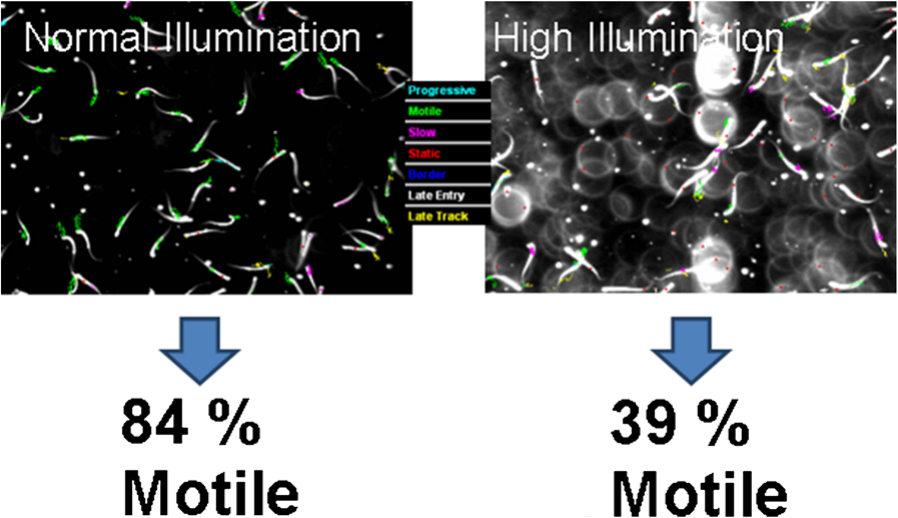
**Analysis of the motility of the same sperm sample with slight changes in instrument settings. **Due to over-illumination dust particles and water droplets are recognized as static sperms which alters the results profoundly.

Furthermore, inadequate adjustments of the experimental settings are to produce false negative or false positive results which may affect the overall outcome of the final study. One example is an inadequate temperature control since slight reductions in temperature can profoundly affect motility parameters. Another example is the illumination, which can be individually adjusted for each single experiment. Over-exposure may result in the recognition of dust particles and water droplets as static sperms and this can affect the results in a profound way (Figure 2): a sperm sample with 84% motile sperms using the correct illumination setting may change to 39% motile sperms due to over-illumination. Similar problems may also occur when using different microscopes (with same software settings) since they are individually susceptive to light intensity and contrast settings. Further problems, which may occur due to inappropriate trained staff, may be the usage of wrong slides (e.g. a 20 μm slide is not sufficient to determine sperm motility due to the limited space). The significant impact of user training is described in detail in an article by Holt and co-workers [9]. Hence, standardization of testing conditions (as possible) adopted for individual computer-assisted sperm analysis combined with a robust validation prior to use in experimental settings is invaluable. We suggest that this validation procedure should at the very least verify reproducibility and accuracy and should also be published in the supplementary information of the respective scientific journal. Furthermore, each individual person who uses the respective CASA apparatus has to be thoroughly trained in order to guarantee the proper usage of CASA. Regularly re-training should be performed. Adopting the doctrine of publishing all instrument settings within a scientific paper would further allow the reader to properly interpret the results, particularly any false positive or false negative data generated by unqualified or poorly-trained staff using specific CASA devices.

Unfortunately, although some general and important guidelines exist for the measurement of sperm motility analysis and the usage of CASA [10] a detailed, generally-accepted guideline for specific internal CASA settings and adjustments of important laboratory species is missing. Obviously, due to specific software algorithms for specific CASA devices (which are able to change the obtained results of specific parameters like amplitude of lateral head displacement (ALH)), it is hardly possible to generate one general guideline with specific CASA settings. However, several important parameters could be standardized for routine analysis studies – even just for individual commonly used machines: e.g. when the cells are slow or progressive (which path velocity (μ/s) or amount of straightness (%) should be considered). Most laboratories have their own settings, which are described in internal standard operating procedures (SOP). This is mostly suitable for internal testing, since each group applies the same settings for each in-house experiment and thereby treatment-related effects can be recognized. However, to compare different studies from different groups, the same settings should be used among all groups using this specific CASA system. This would help to understand the whole picture in contrast to look only on a small puzzle piece. Furthermore, in order to rapidly spread internationally advised CASA settings, publications should only be accepted by scientific journals when these standards are adopted or, if scientifically not possible, the complete settings should be published in the supplementary information.

Finally it is important to note, that of course not only instrument settings need to be harmonized but also the experimental conditions like culture media, incubation times in media, etc. These parameters do also have significant impact on sperm quality.

## Discussion and conclusion

Three key messages can be drawn which should be taken into consideration whenever working with CASA devices:

1) An international working group should define minimum standard settings for routine measurements of sperms for each important individual laboratory species and most commonly used CASA systems.

2) Whenever these routine measurements cannot be used (due to specific experimental conditions) the whole list of internal settings should be published in the supplementary information of the respective journal or in the appendix of the report.

3) Extensive validation procedures should take place before using CASA in an experiment in order to avoid wrong settings which may lead to false positive or false negative results.

## Abbreviations

CASA: Computer assisted sperm analysis; ALH: Amplitude of lateral head displacement; SOP: Standard operating procedure.

## Competing interests

BSL Bioservice Scientific Laboratories GmbH is a contract research organization serving a number of industries and conducting experimental studies, including computer-assisted sperm analysis. However, no competing interest exists which is directly related to this article or which may influence the scientific integrity of this article.

## Authors’ contributions

SCC: Planed and performed the experiments and drafted the article. ALL: Revised the manuscript critically for important intellectual content. Both authors read and approved the final manuscript.
